# General anaesthesia-related complications of gut motility with a focus on cholinergic mechanisms, TRP channels and visceral pain

**DOI:** 10.3389/fphys.2023.1174655

**Published:** 2023-05-18

**Authors:** Alexander V. Zholos, Dariia O. Dryn, Mariia I. Melnyk

**Affiliations:** ^1^ ESC “Institute of Biology and Medicine”, Taras Shevchenko National University of Kyiv, Kyiv, Ukraine; ^2^ O.O. Bogomoletz Institute of Physiology, National Academy of Sciences of Ukraine, Kyiv, Ukraine

**Keywords:** smooth muscles, DRG neurons, visceral pain/visceral nociception/visceral hypersensitivity, TRP channels, G proteins, neurotransmission, intracellular calcium, patch-clamp

## Abstract

General anesthesia produces multiple side effects. Notably, it temporarily impairs gastrointestinal motility following surgery and causes the so-called postoperative ileus (POI), a multifactorial and complex condition that develops secondary to neuromuscular failure and mainly affects the small intestine. There are currently limited medication options for POI, reflecting a lack of comprehensive understanding of the mechanisms involved in this complex condition. Notably, although acetylcholine is one of the major neurotransmitters initiating excitation-contraction coupling in the gut, cholinergic stimulation by prokinetic drugs is not very efficient in case of POI. Acetylcholine when released from excitatory motoneurones of the enteric nervous system binds to and activates M2 and M3 types of muscarinic receptors in smooth muscle myocytes. Downstream of these G protein-coupled receptors, muscarinic cation TRPC4 channels act as the major focal point of receptor-mediated signal integration, causing membrane depolarisation accompanied by action potential discharge and calcium influx via L-type Ca^2+^ channels for myocyte contraction. We have recently found that both inhalation (isoflurane) and intravenous (ketamine) anesthetics significantly inhibit this muscarinic cation current (termed *mI*_*CAT*_) in ileal myocytes, even when G proteins are activated directly by intracellular GTPγS, i.e., bypassing muscarinic receptors. Here we aim to summarize Transient Receptor Potential channels and calcium signalling-related aspects of the cholinergic mechanisms in the gut and visceral pain, discuss exactly how these may be negatively impacted by general anaesthetics, while proposing the receptor-operated TRPC4 channel as a novel molecular target for the treatment of POI.

## 1 Introduction

Postoperative ileus (POI) remains one of the most common and costly for the healthcare system complications of surgery, in particular abdominal surgery. POI is a multifactorial problem, whereby administration of general anaesthetics and anxiolytics is certainly one of the multiple risk factors, and is likely especially important during the first neurological phase of POI involving sympathetic and enteric nervous systems (Venara et al., 2016; Wattchow et al., 2021).

General anaesthetics are primarily aimed at targeting specific receptors of the central nervous system (CNS), but these drugs can also affect other molecular off-targets, such as receptors and ion channels outside of the CNS. Understanding how general anaesthetics can negatively affect gut motility would seem to require, in the first place, knowledge of their effects within the enteric nervous system (ENS), as well as on the pacemaker cells of the gastrointestinal (GI) tract - Interstitial Cells of Cajal (ICC cells), which coordinate and regulate the sensory, secretory and motor functions of the gut (Spencer and Hu, 2020). Indeed, one of the pioneer studies in this area has demonstrated that phencyclidine and related drugs including ketamine decreased neurogenic contractions of guinea-pig ileum and shifted the dose-response curve to acetylcholine to higher agonist concentrations. The authors have thus suggested that these drugs interact with both enteric neurones and smooth muscle myocytes (Gintzler et al., 1982). However, there has not been much subsequent progress towards delineating specific molecular and/or cellular pathways involved. On the other hand, among various prokinetic drugs used for POI treatment (Venara et al., 2016), low concentrations of neostigmine, which is an inhibitor of acetylcholinesterase, may be beneficial (results of the recent systematic review and meta-analysis performed by Liao et al., 2021), which calls for a better understanding of the possible dysfunction of the acetylcholine-mediated signal transduction during the impairment of GI motility by general anaesthetics.

Strong evidence has been accumulated over the recent years indicating a major functional role of various subtypes of Transient Receptor Potential (TRP) channels in smooth muscles and sensory neurons (Venkatachalam and Montell, 2007; Tsvilovskyy et al., 2009). Recent studies (Matta et al., 2008; Abeele et al., 2013; Dryn et al., 2018; Wang et al., 2019; Melnyk et al., 2020) have shown that TRP channels could interact with general anesthetics at subclinical doses, which makes them highly likely primary candidates for the development of side effects produced by local and general anaesthetics. Thus, in recent years we focused our research on the problem of how general anaesthetics, such as isoflurane and ketamine, affect acetylcholine-activated TRPC4 channels, which mediate muscarinic cation current in ileal myocytes, termed mI_CAT_ (Tsvilovskyy et al., 2009). Since TRPC4 channels are widely expressed in the CNS, ENS, ICC and GI smooth muscle myocytes they are increasingly proposed as promising pharmacological targets (Boesmans et al., 2011), but their role in GI pathophysiology in general, and specifically in the pathogenesis of POI, remains to be better elucidated, prompting us to summarize the current status of this research in this Perspective.

### 1.1 TRP channels and pain

Chronic pain significantly impairs quality of life. As reviewed by Julius (2013), TRP channels play a significant role in pain signalling (Julius, 2013). TRP channels are known as polymodal sensors of various stimuli, including chemical modulators, reactive oxygen species, changes in pH and temperature, and mechanical forces (Wu et al., 2010). Being a vital protective mechanism, pain perception could be a considerable problem under some pathological conditions, such as inflammation, when chronic pain develops. Among multiple other co-morbidities, severe pain also correlates with POI. Recent studies have highlighted the involvement of several members of the TRP superfamily of ion channels in producing pain. The cold receptor TRPM8 is expressed in various sensory neurons and is involved in cold nociception (Knowlton et al., 2013). Notably, these channels perceive noxious cold, innocuous cooling and TRPM8-mediated analgesia differently (Laing and Dhaka, 2016). These channels are implicated in inflammatory and neuropathic cold allodynia and other cold hypersensitivity (Bautista et al., 2007; Colburn et al., 2007; Moran and Szallasi, 2018). A recent study has identified the efficacy of novel TRPM8 antagonists in treating both inflammatory and neuropathic pain (De Caro et al., 2018). Different pharmaceutical companies have been developing novel TRPM8 antagonists as pharmacological treatment for chronic or inflammatory pain, migraine and chemotherapeutic-induced allodynia (Weyer and Lehto, 2017). However, as of yet there are no ongoing clinical trials of these compounds.

The other member of the TRP superfamily which is expressed in neuronal, smooth muscles cells and other non-neuronal cells is TRPV4, a multimodal sensor which underlies regulation of several important physiological functions such as osmotic, mechanical and warm temperature sensation (Everaerts et al., 2010; Moore et al., 2018). Recent studies revealed the involvement of this channel in nociception, in particular in joint- and skin-mediated inflammatory pain, neuropathic pain and visceral pain (Alessandri-Haber et al., 2004; Brierley et al., 2008; Moore et al., 2013; O’Conor et al., 2016; Qu et al., 2016). These data indicate that TRP channels are promising therapeutic targets for chronic pain relief. Better understanding of the underlying molecular mechanisms of the TRP channels’ role in nociception could promote the search for chemical compounds as prospective and novel pharmacological approaches targeting these channels for effective pain relief.

### 1.2 The role of TRP channels in visceral pain generation triggered by smooth muscle spasm

The GI tract has differentiated sensory afferent innervation, with sensory neurones located in the dorsal root ganglia (DRGs), nodose ganglia and the inferior ganglion of the vagus nerve (Brookes et al., 2013; Spencer and Hu, 2020). Visceral pain can be associated with smooth muscle (SM) spasms, in turn causing irritable bowel syndrome (IBS). Abdominal pain is a primary symptom of IBS (Keszthelyi et al., 2012a; 2012b). Several of the TRP channels (most notably TRPA1, TRPC4, TRPV1 and TRPV4) are expressed in the gut, where they play important roles in multiple pathophysiological processes, including visceral nociception and pain (Boesmans et al., 2011; Holzer, 2011). Thus, TRPC4 channels opened secondary to muscarinic acetylcholine receptor activation trigger excitation and contraction of small intestinal smooth muscles (Bolton et al., 1999; Tsvilovskyy et al., 2009). TRPV4 senses local pressure that can become painful when it exceeds certain threshold level (Liedtke, 2005). Moreover, TRPV4 in afferent nerves can be sensitized *via* protease-activated receptor 2 thus evoking visceral hyperalgesia (Sipe et al., 2008). The molecular pathophysiology of IBS is not completely understood (Shah et al., 2020), but among other ion channels, TRPV4 and TRPA1 have been suggested as important sensory channels in IBS (Yang et al., 2022).

Currently, three main agent classes are used for SM antispasmodic action: antimuscarinic agents (Tobin et al., 2009), calcium channel inhibitors (Evangelista, 2004), and direct smooth muscle relaxants (Subissi et al., 1983), but their efficiency is less than optimal, making these compounds questionable for their clinical use. There is thus an urgent need to develop other therapies for treating chronic abdominal pain, such as TRP channels modulators.

### 1.3 The mechanism of general anaesthesia action on TRP channels

Inhaled anesthetics rapidly equilibrate between air in the alveoli and capillary blood. Small hydrophobic molecules like anesthetics can first of all affect the membrane lipid bilayer, thus nonspecifically altering the functions of multiple, if not all, transmembrane proteins (Tsuchiya and Mizogami, 2013). The second, much better understood group of their molecular targets includes plasma membrane receptors and ion channels. Among the latter, both voltage-gated (Ca^2+^, Na^+^ and K^+^ channels of different types) and ligand-activated (such as nicotinic acetylcholine receptors, serotonin, glycine and GABA_A_ receptors) received considerable attention in this context (Jenkins et al., 1996; Tassonyi et al., 2002; Hara and Sata, 2007; Alberola-Die et al., 2011; Germann et al., 2016; Liu et al., 2019; Mathie et al., 2021). For example, the inhalational anaesthetic isoflurane, which is a halogenated ether, targets GABA_A_, glutamate and glycine receptors, while ketamine is best characterised as an antagonist of NMDA receptors, which determines its strong analgesic action (Franks, 2006; Franks, 2008).

The TRP channels discussed above represent yet another group of such targets. There is indeed growing evidence in this area of research showing that, for example, halothane, chloroform, and propofol can inhibit TRPC5 channels (Bahnasi et al., 2008), isoflurane can activate TRPA1 channels in sensory DRG neurones (Matta et al., 2008), while propofol affects TRPA1 and TRPV1 channels as became evident from observing propofol-induced vasorelaxation of coronary arterioles (Wang et al., 2015).

**FIGURE 1:**
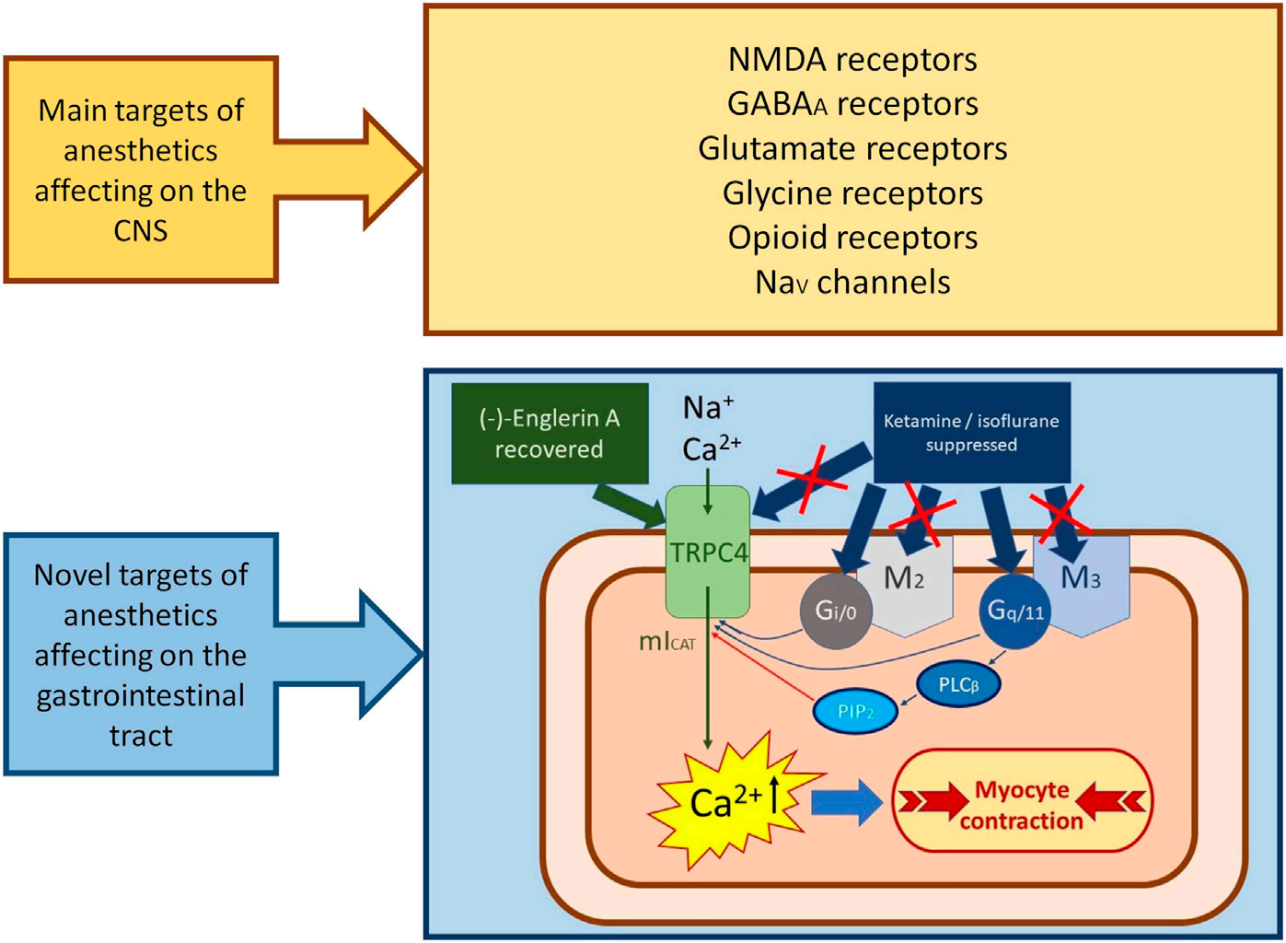
The schematic illustration of the possible mechanism of anaesthetics side-effects action on the novel molecular targets in intestinal myocytes. Acting in synergy with muscarinic M2 and M3 receptors coupled to G_i/o_- and G_q/11_-proteins, respectively, TRPC4 channels are the main molecular component of depolarizing inward current mI_CAT,_ which is the principal regulator of cholinergic excitation-contraction coupling in intestinal smooth muscles. Anaesthetics (ketamine and isoflurane) strongly suppressed *mI*_*CAT*_, acting more likely on G-proteins, but minimally on M2/M3 receptors. TRPC4 channels direct agonist (−)-englerin A recovered ketamine-induced inhibition of mI_CAT_, suggesting that this anaesthetic is targeting G-proteins rather than TRPC4 channels.

Acetylcholine, a major neurotransmitter that plays multiple important roles in the central and peripheral nervous system, activates muscarinic acetylcholine receptors (mainly of M2 and M3 subtypes), which are the main excitatory receptor subtypes expressed in GI smooth muscles (Bolton, 1972; 1979; Zholos, 2006). This, in turn, results in the openings of two receptor-operated cation channels, TRPC4 and TRPC6, of which TRPC4 is of main importance since it mediates about 85% of mI_CAT_ (Tsvilovskyy et al., 2009). Signal transduction pathways leading to TRPC4 are complex, since two receptor subtypes, which are differentially coupled to Gi/o and Gq/11 proteins, are involved (M2 and M3 receptors, respectively). These have been previously extensively studied and reviewed (Zholos and Bolton, 1997; Bolton et al., 1999; Zholos et al., 2004; Zholos, 2006; Sakamoto et al., 2007; Tanahashi et al., 2020), as summarised schematically in Figure 1. In brief, these receptors systems act in synergy, whereby the M_2_/G_i/o_ is of primary nature, while the M_3_/G_q_/PLC system and the increase in intracellular Ca^2+^ concentration it produces by InsP_3_-evoked Ca^2+^ release play both permissive (at least in part via PIP_2_ depletion) and potentiating (via [Ca^2+^]_i_ elevation) roles. We therefore reasoned that inhibition of mI_CAT_ generation as the primary mechanism of cholinergic excitation-contraction coupling in the gut (Bolton et al., 1999) can occur at different levels, ranging from muscarinic receptors and the G-proteins that are coupled to them, and to Ca^2+^ signalling and TRPC4 channels themselves.

We have addressed these possibilities using a range of experimental techniques, from patch-clamp recordings to *in vitro* contractile recordings, in our recent studies of isoflurane and ketamine effects on mI_CAT_ and spontaneous as well as carbachol-stimulated contractions of ileal smooth muscles (Dryn et al., 2018; Melnyk et al., 2020). To bypass the receptor activation step, GTPγS infusion via patch-pipette was employed for direct activation of all trimeric G-proteins. Intriguingly, both isoflurane and ketamine strongly inhibited both carbachol- and GTPγS-induced mI_CAT_ at clinically relevant concentrations, and the inhibitory effects had much in common. Thus, muscarinic receptors are not the major targets of their action, and hence any strategy aimed at the upregulation of mACh receptor activity, such as inhibitors of acetylcholinesterase, would be, in theory, not very efficient. At the same time the effect of ketamine was effectively opposed by the direct TRPC4 agonist (−)-englerin A (EA) indicating that the function of the channel itself was preserved (Melnyk et al., 2020) (Figure 1). We thus concluded that TRPC4 agonists may be used for the correction of GI motility suppression induced by general anesthesia. It is worth noting that there has recently been significant progress in developing nontoxic analogue of EA (Seenadera et al., 2022).

### 1.4 Post-traumatic stress disorder and antidepressants

Currently, the most effective and widely used treatments for post-traumatic stress disorder (PTSD) are antidepressants and anxiolytics, but other novel treatments are being considered, especially for the treatment of more refractory and disabling cases of PTSD (Ipser and Stein, 2012; Liriano et al., 2019). Thus, ketamine is not only a widely used anesthetic, but has recently been considered as a potential antidepressant (Duman et al., 2012; Zanos and Gould, 2018; Jeong et al., 2022), in particular as a promising and novel pharmacotherapeutic agent for PTSD patients, especially in more complex cases (Liriano et al., 2019).

Recent studies have shown that some novel modulators of TRP channels possess antidepressant action. A recently identified inhibitor of TRPC4/C5 channels, M084, has demonstrated antidepressant and anxiolytic effects (Yang et al., 2015). HC-070, a new small molecule antagonist of these channels, also possess antidepressant effects and may be proposed as a treatment for a number of symptoms of psychiatric disorders (Just et al., 2018). Antidepressants, in turn, affect TRP channels, and not only in the nervous system, but also in other systems and organs, where they can produce side effects. It was shown that tricyclic antidepressants, which are also used for treating IBS, inhibited TRPC4 channels in colonic myocytes, resulting in suppression of GI motility (Jeong et al., 2022). These results well correlate with our recent studies of the inhibitory action of ketamine and isoflurane on the TRPC4 channel-mediated mI_CAT_ in mouse small intestinal myocytes (Dryn et al., 2018; Melnyk et al., 2020). Thus, antidepressants and anxiolytics can modulate the TRP channels function, which in turn could lead to intestinal complications.

## 2 Conclusion and further research

Currently, continuation of the studies of the side effects of anaesthetics and antidepressants on TRP channels, in particular in intestinal myocytes, remains an important task. War and military actions are the most significant factors of PTSD development, and also of the increase in the number and complexity of surgical interventions. According to the latest medical reports from the Ministry of Health of Ukraine, as a result of the Russian invasion of Ukraine in 2022 and the ongoing war, the risk of PTSD occurrence can be very high and will amount from 4.5 to 15 million people, including both military personnel and civilians (Bryant et al., 2022; Cai et al., 2022; Chaaya et al., 2022; Zaliska et al., 2022). The results of such studies as outlined in this Perspective can propose some recommendations for optimizing protocols for the use and dosage of certain types of anaesthetics and antidepressants in medical practice, in particular in the treatment of PTSD. Thus, our own future studies will be aimed at revealing ion channel mechanisms of side effects of other widely used anaesthetics, as well as anxiolytics and antidepressants, and their combinations. Moreover, other members of the TRP family, in particular TRPV4 and TRPM8 channels, which are important for the regulation of blood vessel tone, need to be more fully characterized in this context (Melanaphy et al., 2016; Dryn et al., 2018). There is accumulating evidence that some anaesthetics can affect these two types of channels (Abeele et al., 2013; Wang et al., 2019), as well as vascular tone (Liu et al., 2009; Gille et al., 2012; Sakai et al., 2014), therefore this research area is also relevant and promising.

## Data Availability

The original contributions presented in the study are included in the article/Supplementary Material, further inquiries can be directed to the corresponding author.

## Abbreviations

CNS, central nervous system; DRG, dorsal root ganglia; EA, (−)-englerin A; ENS, enteric nervous system; GI, gastrointestinal; IBS, irritable bowel syndrome; mAChR, muscarinic acetylcholine receptor; mI_CAT,_ muscarinic receptor cation current; POI, postoperative ileus; PTSD, post-traumatic stress disorder; SM, smooth muscle; TRP, Transient Receptor Potential.
